# Iodine increases and predicts incidence of thyroiditis in NOD mice: Histopathological and ultrastructural study

**DOI:** 10.3892/etm.2012.826

**Published:** 2012-11-22

**Authors:** STELLA MARIA PEDROSSIAN VECCHIATTI, MARIA LUISA GUZZO, ELIA GARCIA CALDINI, HÉLIO BISI, ADHEMAR LONGATTO-FILHO, CHIN JIA LIN

**Affiliations:** 1 Department of Pathology, University of São Paulo School of Medicine, University of São Paulo, São Paulo, Brazil;; 2 Endocrinology Service of Municipal Hospital of São Paulo, University of São Paulo, São Paulo, Brazil;; 3 Bioterism Center of University of São Paulo School of Medicine, University of São Paulo, São Paulo, Brazil;; 4 Laboratory of Medical Investigation (LIM) 14, Faculty of Medicine, University of São Paulo, São Paulo, Brazil;; 5 Life and Health Sciences Research Institute (ICVS), School of Health Sciences, University of Minho, Braga;; 6 ICVS/3B’s - PT Government Associate Laboratory, Braga,Guimarães, Portugal;; 7 Molecular Oncology Research Center, Barretos Cancer Hospital, São Paulo, Brazil

**Keywords:** iodine, thyroiditis, lymphocytic infiltration, non-obese diabetic mice, autoimmunity, experimental autoimmune thyroiditis, iodine-induced thyroiditis

## Abstract

Prolonged intake of large amounts of iodine has been reported to increase the incidence of hypothyroidism in humans, as well as in animals which are prone to spontaneously developing autoimmune thyroiditis. We sought to investigate the histopathological consequences of large amounts of dietary iodine on the thyroid gland and observe the occurrence of lymphocytic infiltration associated with the time of exposure to iodine. An experimental model using non-obese diabetic (NOD) mice was analyzed. A potassium iodide intake of 0.2 mg/animal/day was administered via drinking water, in experimental groups of 60 and 90 days (EG60 and EG90). Distended rough endoplasmic reticulum, degenerated mitochondria, debris and amorphous spaces or ‘ill-defined’ spaces were observed with electron microscopy (EM). Lymphocyte infiltration was observed in the two groups and the time of exposure to iodine did not increase the appearance of lymphocyte infiltration but significantly associated with the development of necrosis. The results of the present study demonstrated that the NOD mouse is a feasible experimental model for thyroiditis induced by iodine administration and may represent an opportunity to analyze the steps and factors associated with genetic autoimmune thyroiditis. High doses of ingested iodine were observed to precdict and increase the incidence of the thyroiditis process.

## Introduction

The global increase in the prevalence and incidence of thyroiditis has been attributed to the introduction of table salt, water or oil iodation to prevent goitre in iodine-deficient areas and is also associated with the elevated urinary iodine observed in non-defficient iodine areas (1–5), which has encouraged the development of a number of experimental studies (6,7). Several sources of iodine induced thyroiditis were correlated with the development of thyroid autoantibodies (8), including iodinated radiological contrasts (9–12) and medical drugs (13). However, the role of iodine in these processes remains controversial and not fully understood.

The initial steps of the autoimmune process in the thyroid are poorly understood. Necrosis of follicular cells is considered to be the crucial event that triggers the autoimmune process (14) and its induction by iodine is well documented (7,15). Thyroid epithelial cells are constantly exposed to reactive oxygen species (ROS) which are physiologically necessary for thyroid hormone synthesis. However, when ROS are produced excessively, they become toxic and induce cellular destruction and inflammation in various models of iodine-induced thyroid involution (7)

The inflammatory autoimmune condition of the thyroid gland depends on numerous factors, presumably including the patient genetic profile which is proposed to be responsible for at least 50% of all autoimmune thyroiditis, although no conclusive genetic factors have been described (16). Environmental factors, such as thiocyanates from tobacco and stress, and endogenous, including pregnancy, have been implicated in its etiopathogeny (16). There is also evidence to support the theory that non-inflammatory processes, such as apoptosis, are important in the destruction of thyroid follicles (7,17–20). Notably, a significant reduction of caspase-3 expression by peripheral T cells in thyroiditis has been demonstrated (21). Certain animals spontaneously develop auto-immune thyroiditis (SAT), such as NOD.H-2h4 mice, BB/W rats, Cornell C and obese strain chickens (OSCs) (6,22,23), and for this reason, the majority have been used in various studies aiming to demonstrate the effects of iodine in induced thyroiditis.

The present study aimed to investigate the effects of oral iodine consumption in non-obese diabetic (NOD) mice in an experimental model tailored to observe the histopathological alterations of the thyroid under light microscopy (LM) and electron microscopy (EM).

## Materials and methods

### Animals and treatment

Matrices of wild-type NOD mice were acquired from the Center for Development of Experimental Models for medicine and Biology (CEDEME) from the University of São Paulo (Brazil) and were maintained at the Animal Care Facility of Faculty of Medicine of São Paulo University (FMUSP). A total of 64 female NOD mice aged between 4 and 6 weeks were selected and divided into 4 groups of equal size. Of these groups, EG60 and EG90 received ∼0.2 mg/animal/day of oral potassium iodine in drinking water while the remaining groups (CG60 and CG90) had no oral supplement of iodine. Mice assigned to the first treatment group (EG60) were supplemented with iodine for 60 days. The second group (EG90) received treatment with iodine for 90 days. The control groups were labeled either CG60 or CG90 depending on whether they were sacrificed after 60 or 90 days, respectively. The animals were fed *ad libitum* with standard commercial food. All experimental procedures were approved by the Ethics Commission for Research Project Analysis (CAPPesq) of FMUSP.

### Specimen preparation

The animals were weighed and anesthetized with intraperitoneal injections of 100 mg/kg ketamine and 10 mg/kg xylazine prior to the thyroidectomy. Euthanasia was performed by cervical dislocation. The thyroid tissues were fixed in buffered formalin (10%) and embedded in paraffin. Sections (3 μm) were stained with hematoxylin and eosin and the slides were mounted with Entellan medium (Merck, Darmstadt, Germany) and analyzed using LM. For EM, the sections of thyroid material were fixed in 2% glutaraldehyde phosphate buffer 0.1 M (pH 7.4) and dissected while immersed in this glutaraldehyde solution at x4 magnification. The first post-fixation step was performed in 1% osmium tetroxide and the second step with 1% uranila overnight. Material dehydration was performed using acetone in a graduated series of 30–100% before being embedded in Araldite resin. Sections (70 nm) were examined using a Jeol 1010 transmission electron mircroscope (TEM) (Tokyo, Japan).

Thyroiditis was defined as mononuclear interstitial infiltration regardless of its intensity.

### Statistical analysis

Pearson’s Chi-squared (χ^2^) test and Fisher’s exact test (when n<5) were used to study the association between iodine treatment and frequency of thyroiditis. P≤0.05 was considered to indicate a statistically significant difference.

## Results

### Body weight

The average weight of the animals in the experimental and control groups was ∼25 g and no evidence of catabolic processes was observed in these animals.

### Development of thyroiditis

Treatment with oral iodine for 60 days was associated with the development of thyroiditis in NOD mice. Of 16 mice in the EG60 group, 8 exhibited thyroiditis with oral iodine ingestion. By contrast, no thyroiditis was observed in the CG60 mice (Table I). These differences in the frequency of mice with thyroiditis were statistically significant (P=0.0012) according to Fisher’s exact test. Oral iodine ingestion for an additional 30 days did not increase the frequency of mice with thyroiditis and 3 animals in the CG90 group exhibited thyroiditis (Table I). The difference in the frequency of thyroiditis between the EG90 and CG90 groups, however, was not statistically significant (P=0.0812). Of the mice assigned to EG90, 1 died of unknown causes.

### Light microscopy findings

**Figure 1.**
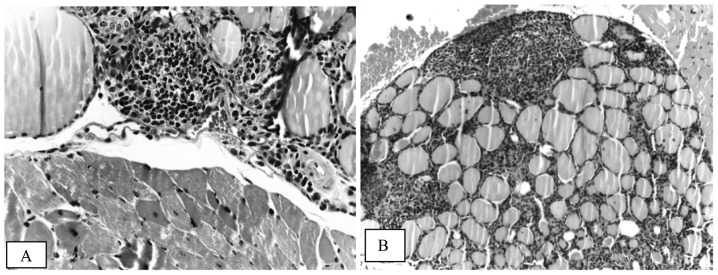
Light microscopy showing (A) extensive and (B) focal lymphocytic infiltration. Notice parathyroid gland embedded in the upper portion of section that corresponds to thyroid

**Figure 2.**
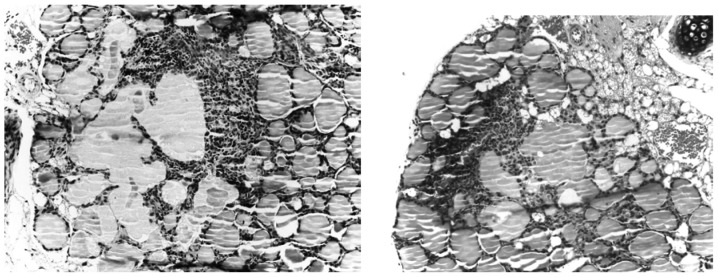
Light microscopy. Lymphocytic infiltration around coalescent follicles and/or cysts.

**Figure 3.**
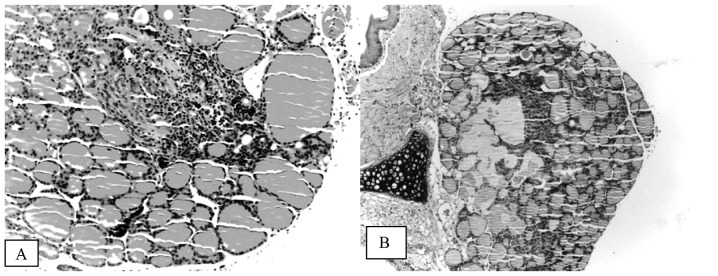
Light microscopy. (A) Necrosis and lymphocytic infiltration. (B) Large follicles with rupture of the basal lamina.

**Figure 4.**
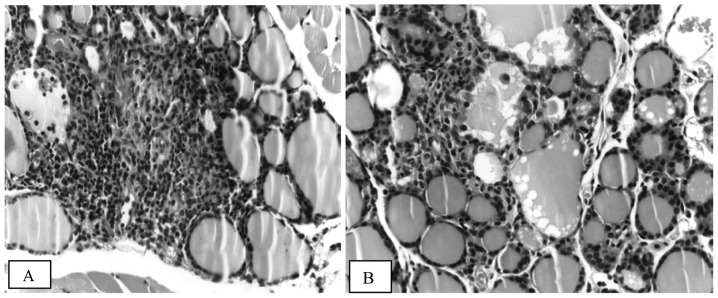
Light microscopy. (A) Macrophages inside follicular lumen and and small follicles around infiltration, (B) plasmocitoid cell inside a follicle.

The most prominent morphological feature characterizing iodine-induced thyroiditis in NOD mice was lymphocyte infiltration which varied from few small foci of invading lymphocytes located in the interstitia of 3 or 4 follicles (Fig. 1A) to the extensive infiltration of a substantial portion of the thyroid lobe (Fig. 1B). The lymphocytic infiltration was repeatedly observed in the vicinity of large follicles or cysts formed by coalescing follicles (Fig. 2) and sometimes coexisted with the necrosis of follicle cells and disruption of thyroid architecture (Fig. 3). Atrophic follicles with reduced diameters were observed surrounded by or in the periphery of a lymphocytic infiltration (Fig. 4). Longer treatment with iodine was associated with a more diffuse and abundant lymphocyte infiltration pattern (data not shown). Similar lymphocyte infiltration was observed in the control mice (3 animals in CG90) that exhibited spontaneous thyroiditis (data not shown).

Focal areas of necrosis were observed in 5 mice of the EG60 group and these were observed concomitantly with thyroiditis in 3 of these animals. In the EG90 group, 1 mouse developed thyroid necrosis without thyroiditis. None of control animals in either the CG60 or the CG90 exhibited necrosis (Table I).

**Figure 5.**
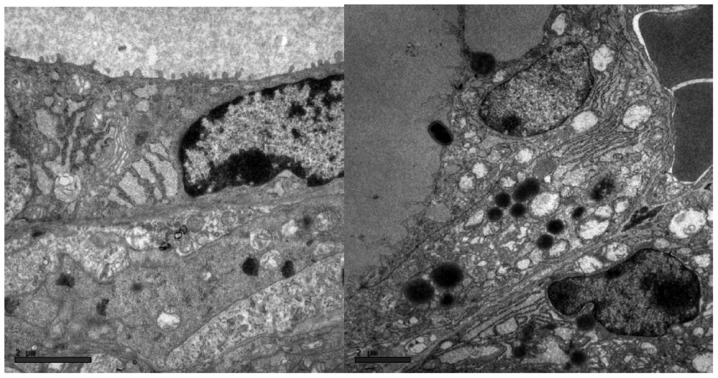
Electron microscopy. Distended rough endoplasmic reticulum and swollen mitochondria.

**Figure 6.**
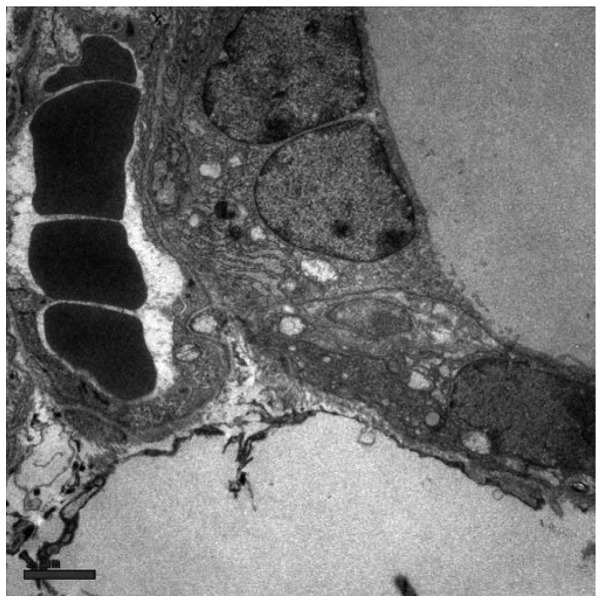
Electron microscopy. Debris and swollen mitochondria.

**Figure 7.**
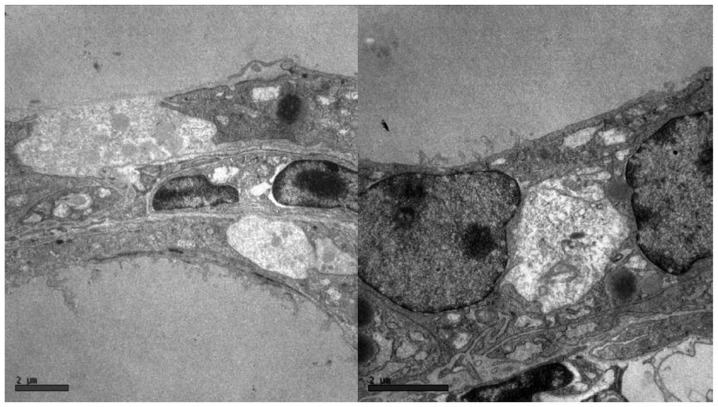
Electron microscopy. Amorphous spaces.

**Figure 8.**
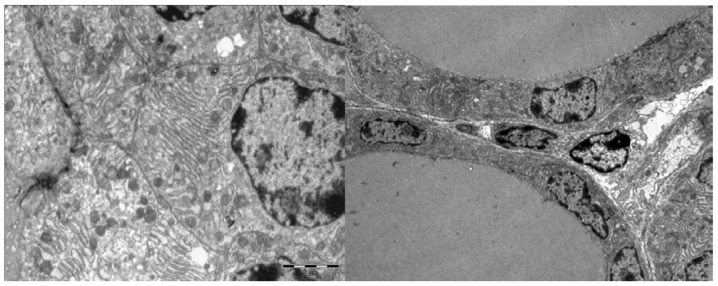
Electron microscopy. Control group: no alterations observed.

The follicle cells in iodine-treated mice (EG60) exhibited distended rough endoplasmic reticulum and swollen, degenerated mitochondria with a loss of cristae (Fig. 5). Other ultrastructural abnormalities included subcellular debris (Fig. 6) and clear, ill-defined spaces where no nuclei or organelles could be identified with certainty (Fig. 7). We named this structure an ‘amorphous space’ due to its unknown nature. Examination of semi-thin sections of the thyroid specimens did not reveal lymphocytic infiltration above the regions where ultrastructural alterations were observed (data not shown). Accordingly, these ultrastructural abnormalities were hypothesised to result from the toxic effects of iodine on thyroid tissue, since they were not observed in the control animals (Fig. 8).

## Discussion

The results presented in the present study partially endorse the hypothesis that iodine has an important involvement in the development of thyroiditis. This may be observed by the comparison of the CG60 group, with no animals affected, with the EG60 group, where 50% of the animals exhibited thyroiditis. Additionally, a higher frequency of thyroiditis was observed in the EG90 group. The etiopathogeny of autoimmune thyroiditis continues to be broadly discussed and salt iodation and elevated urinary iodine are frequently suggested as factors responsible for inducing autoimmune thyroiditis (3–5). The results of the present study support this theory since the control animals did not develop thyroiditis except at the reported incidence of NOD mice (24).

Notably, a longer exposure to iodine did not appear to increase the frequency of thyroiditis. This suggests that the iodine is a stochastic rather than deterministic factor for the development of thyroidal autoimmunity and that iodine may trigger an earlier appearance of thyroid autoimmune process. We propose that the ultrastructural alterations triggered by treatment with iodine represent early signs of follicular cell lesions which contribute to exposing the immune system to thyroidal autoantigens, thus inducing immune cell infiltration.

The incidence of 18.7% for lymphocytic infiltration in the CG90 group is quite similar to that reported by Damotte *et al*(24) (14.3%) using the same mouse lineage. These authors also observed that the first lymphocytic infiltration in the wild-type NOD mice occurred within 14–15 weeks, but predominantly in the male colony.

Similar to the human thyroiditis, the mice also exhibited lymphocyte infiltration as the most prominent observation. Although we were able to identify some plasma cell-like lymphocytes (Fig. 4), in general the infiltrating cells in mouse thyroiditis were not predominantly composed of plasma cells, as usually can occur in human thyroiditis. The general appearance of the experimental group EG90 appeared to exhibit increased severity of lymphocytic infiltration compared with EG60, which may be explained, in part, by the evolution of inflammatory process.

The frequency of necrosis observed in the EG60 group (31.25%), is in agreement with the toxic effect of iodine in thyroid cells, as reported by Bagchi *et al*(23). As anticipated, other investigators have described necrosis as the first step to antigen exposure in the autoimmune thyroiditis process (14,23,25). Of note, the frequency of necrosis decreased in the EG90 mice. It is possible that the toxic effects of iodine cannot be sustained for a prolonged period of time or may evoke a desensitization response following repeated exposure. A short-lived toxic effect of iodine may explain why necrosis was considerably less frequent following 90 days of treatment. The coexistence of necrosis and thyroiditis possibly indicates that necrosis triggers the thyroid autoimmunity as suggested by Many *et al*(14). We propose that acute iodine induces blood flow restraint, which contributes to the necrosis of follicular cells, resulting in the inflammatory process and release of thyroid antigens to immune cells.

EM showed alterations compatible with cell degeneration. Distended rough endoplasmatic reticulum and mitochondrial lesions with a loss of cristae were described by Nakazawa *et al*(13) in humans following the use of amiodarone for 19 days. Since no subcellular lesions were observed in the control groups (CG60 and CG90), it is possible that the ultra-structural degeneration is an iodine-induced event.

The amorphous spaces documented in the experimental group have not been reported previously. There is no evidence concerning the spaces’ nature and no reasonable explanation for their occurrence may be proposed currently. However, the distribution pattern suggests that these spaces are not likely to be artefacts. The general appearance of these structures mimics the light cells usually detected in normal and pathological tissues, but unlike light cells no nuclei were observed. Further studies should be considered to test the reproducibility of these findings and their significance. Although unspecific, the cellular debris and mitochondrial lesions, even without lymphocytic infiltration, appear to be related to iodine and the beginning of this autoimmune process.

## Figures and Tables

**Table I.** Summary of histological alterations during the time.

Group	Exposure period	n	Thyroiditis	Necrosis	Relative risk
EG60	60 days	16	8	5	17.00 (1.06–271.78)
EG90	90 days	15^a^	7	1	2.49 (0.78–7.89)
CG60	60 days	16	0	0	-
CG90	90 days	16	3	0	-

a One mouse succumbed to unknown causes during study period.
